# QT Prolongation Complicated with Torsades de Pointes in Prosthetic Mitral Valve Endocarditis: A Case Report

**DOI:** 10.1155/2012/574923

**Published:** 2012-10-04

**Authors:** A. Tounsi, L. Abid, M. Akrout, M. Hentati, S. Kammoun

**Affiliations:** Cardiology Department, Hedi Chaker University Hospital, Sfax 3029, Tunisia

## Abstract

We present the case of a 49-year-old male patient with prosthetic mitral valve endocarditis associated with QT prolongation and torsades de pointes. He was asymptomatic until the end of January 2012, when he was admitted to our hospital emergency unit because of syncope, fever, and suspicion of endocarditis. Cardiologic evaluation was requested and the transthoracic (TTE) and transesophageal (TEE) echocardiograms revealed vegetations on the prosthetic mitral valve. All cultures were positive for methicillin-sensitive *Staphylococcus aureus*. The corrected QT (QTc) interval was markedly prolonged upon admission (QTc 540 ms). He experienced torsades de pointes (TdP) several times and he was recovered after bystander cardiopulmonary resuscitation. The clinical course and the long QTc interval with deep inverted T wave were completely normalized 4 weeks after. He continued on triple antibiotic therapy for 45 days with a good revolution. The clinical features and the possible mechanisms of QT prolongation (inflammation, infection) of this patient are discussed.

## 1. Introduction 

Infective endocarditis (IE) is lethal if not aggressively treated with antibiotics, combined or not with surgery. Developments in antibacterial therapy, clinical microbiology, cardiac imaging, and cardiac surgery have revolutionized its diagnosis and prognosis. The classical fever of unknown origin presentation represents a minority of IE cases today; thus, clinicians need to have a high index of suspicion in unusual presentations [1]. Torsade de pointe (TdP) is a devastating form of polymorphic ventricular arrhythmia associated with correctedQT (QTc) interval prolongation. A few cases of infective endocarditis-induced QTc prolongation complicated by TdP have been reported. In this paper we report a case of prosthetic mitral valve endocarditis complicated with QTc prolongation and recurrent TdP, and ventricular fibrillation.

## 2. Case Report

 A 49-year-old man was admitted to our hospital complaining of intermittent fever and syncope. He had prosthetic mitral valve replacement in 1998.

**Figure 1:**
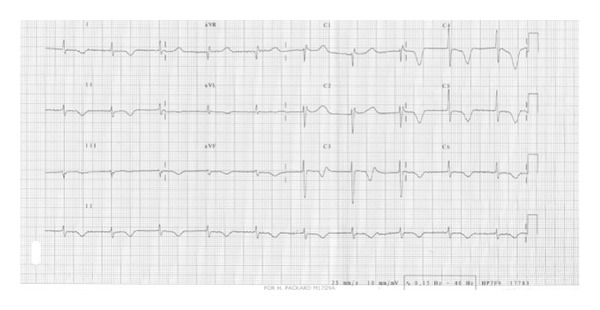
The electrocardiogram showed atrial fibrillation. The QT interval was markedly prolonged.

On physical examination the patient had a fever of 38°C. His blood pressure was 105/70 mmHg. There were no signs of heart failure. He had no signs of splinter haemorrhages, skin nodules, enlarged lymph nodes, nor hepatosplenomegaly. The electrocardiogram showed atrial fibrillation. The QT interval was markedly prolonged (Figure 1). His blood chemistry on admission showed an elevated C-reactive protein (CRP). His leukocyte count on admission and throughout the hospital course was within the normal range. Serum potassium, magnesium, calcium, and thyroid function tests were within the normal range. He had no history of taking drugs that prolong the QT interval. Transthoracic echocardiography revealed a functioning prosthetic bileaflet mitral valve. The mean pressure gradient across the mitral valve was 5. The left atrium was enlarged (62 mm).

**Figure 2:**
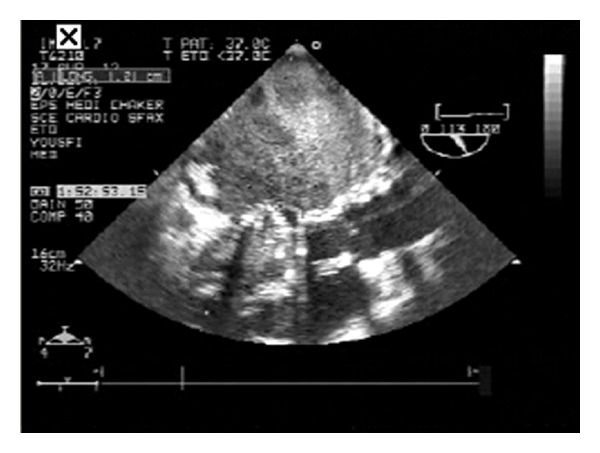
Transesophageal echocardiography (TEE) revealed vegetation on the anterior mitral annulus (1.2 ∗ 0.5 cm).

Because of high clinical suspicion, transesophageal echocardiography (TEE) was done, which revealed vegetation on the anterior mitral annulus (1.2 ∗ 0.5 cm) (Figure 2). A diagnosis of IE was made and an empirical antibiotic regimen of vancomycin 2 g/day, gentamycin 160 mg/day, and rifampicin 1200 mg/day was started on the first day of admission. Cultures were positive for methicillin-sensitive *Staphylococcus aureus.* So the antibiotic regimen was changed to oxacillin (2 g ∗ 6/day), rifampicin (600 mg/day), and gentamycin (160 mg/day).

**Figure 3:**
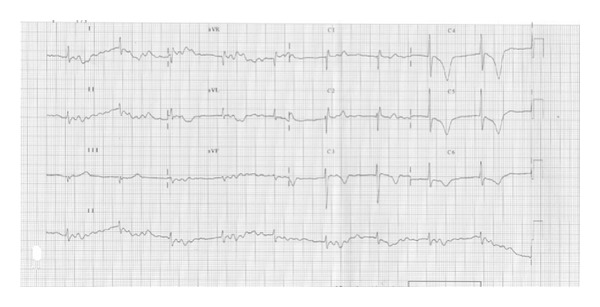
ECG showed bradyarrhythmia, a HR of 48 beats/min, QTc interval prolongation (610 ms), and deep inverted T wave.

**Figure 4:**
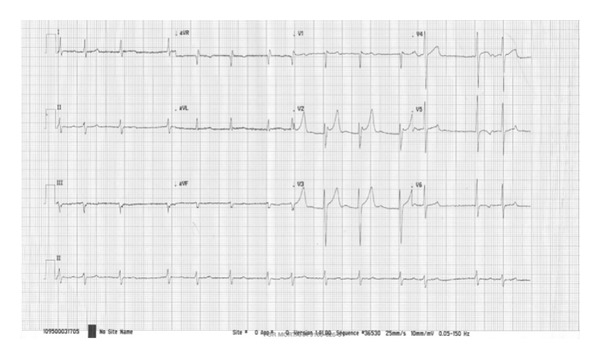
QTc was normalized to 390 ms after day 30.

**Figure 5:**
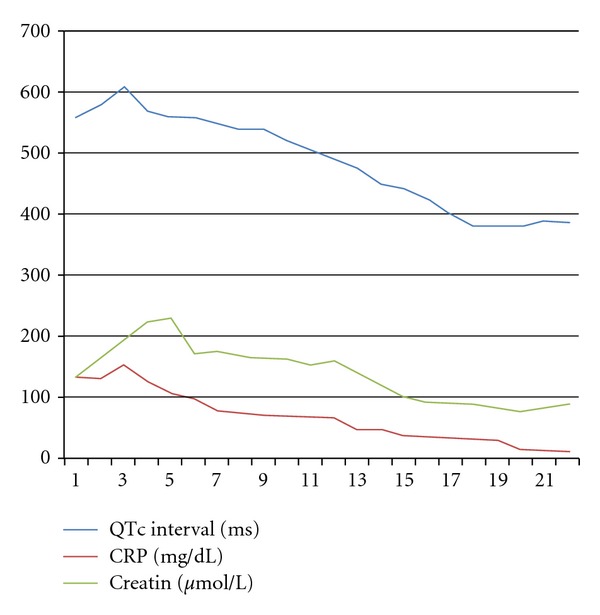
Biological course: CRP: C-reactive protein, Creat: creatinine levels.

On the third day of admission, the patient lost consciousness, ceased spontaneous breathing, BP was unmeasurable, the SpO2 was 36%, and the ECG showed ventricular fibrillation. A direct current of 200 J was applied 3 times using a biphasic defibrillator. The ECG showed resolution of the ventricular fibrillation. However, shortly thereafter TdP-type polymorphic ventricular tachycardia was developed. Defibrillation was performed 3 times, and 1 mg epinephrine and 100 mg lidocaine were intravenously administered. Additionally, magnesium sulfate 2 g was given intravenously over 10 min. The BP increased to 146/80 mmHg and the SpO2 to 95%. The patient was moved to the intensive care unit (ICU) with endotracheal intubation in a stable condition. An ECG showed bradyarrhythmia, a HR of 48 beats/min, QTc interval prolongation (610 ms), and deep inverted T wave (Figure 3). Isoproterenol was continuously administered intravenously at a rate of 3 *μ*g/min. Then, a temporary pacemaker was inserted in the right ventricle apex via the right femoral vein. An echocardiogram showed no remarkable change of left ventricular systolic function. The vital signs stabilized. All electrolytes were within normal limits including potassium (4.1 mmol/L), magnesium (2.2 mg/dL), and calcium (2.45 mmol/L). The patient was moved to the general ward on the 3rd day without relapse of TdP. He did not have any recurrence of arrhythmia and QTc was normalized to 390 ms after day 30 of antimicrobial therapy (Figure 4). He was clinically and hemodynamically stabilized. C-reactive protein decreased to 13 mg/L (Figure 5). He continued on twin antibiotic therapy for 45 days. The patient has remained symptom- and arythmia-free over a 6-month followup.

## 3. Discussion

 Long QT syndrome is an arrhythmogenic cardiovascular disorder with prolongation of ventricular repolarization, including a QTc interval prolongation longer than 440 ms for males or 460 ms for females, and an abnormal T wave (broad, notched, small) that causes ventricular tachycardia such as TdP and ventricular fibrillation resulting in syncope or sudden death [2]. Long QT syndrome has traditionally been categorized as either congenital or acquired. Congenital Long QT syndrome is a heritable ion channel disease due to gene mutations of the transmembrane sodium or potassium ion channel proteins. Acquired factors leading to long QT syndrome include hypokalaemia, hypomagnesaemia, hypocalcaemia marked bradycardia, cocaine abuse, organophosphorus compound poisoning, subarachnoid haemorrhage, stroke, myocardial ischaemia, protein-sparing fasting by using liquid protein diets, autonomic neuropathy, and human immunodeficiency virus disease [3]. Certain common antibiotics, such as erythromycin, appetite suppressants, and decongestants, may trigger dangerous heart rhythms. Antiarrhythmic drugs, calcium channel blockers, psychiatric drugs, antihistamines, antimicrobial and antimalarial drugs, serotonin agonists/antagonists, immunosuppressant, and antidiuretic hormones are capable of prolonging the QT interval [4].

A clinical study on acute promyelocytic leukemia therapy by arsenic trioxide revealed that mild and moderate anaemia did not affect QTc and QTd [5]. Neither hypoproteinaemia nor hyperbilirubinaemia correlated with a prolonged QT interval [6]. However, patients with chronic renal disease have elongated a QT interval, which was further prolonged by dialysis therapy [7]. However, it has recently been suggested that acquired Long QT syndrome is linked to a silent mutation in the gene that causes congenital long QT syndrome [8]. Long QT syndrome can also be divided into adrenergic- and pause-dependent types. 

Most acquired long QT syndrome is the pause-dependent type that causes QTc interval prolongation and arrhythmia via bradyarrhythmia or sinus pause.

Transmural dispersion (i.e., significant differences in repolarization in the various layers of the myocardium) is a feature of TdP and ventricular fibrillation in patients with long QT syndrome [9, 10]. A decrease in the outward potassium ion current or an increase in the slow calcium ion inward current during repolarization resulting from mutation of the gene encoding the ion channel prolongs the action potential duration that leads to early after depolarization (EAD). In addition to the reentry mechanism, EAD and transmural dispersion induce TdP [11]. The mechanisms underlying QTc interval prolongation after conversion to a sinus rhythm are not clearly understood. In the present patient, a hereditary basis of long QT syndrome might be missing as the familial history was scanty; however, a genetic screening test was not conducted. Laboratory tests ruled out the possibility of hypomagnesaemia, hypocalcaemia, or hypokalaemia. Thus, predisposing factors for acquired long QT syndrome may include mitral valve regurgitation caused by infective endocarditis, renal failure, glomerulonephritis, bradyarrhythmia (atrial fibrillation with slow ventricular response), febrile infection [12], and medications, such as diuretics, or antibiotics.

Magnesium sulfate is the treatment of choice for TdP and lidocaine can also be used. Serum potassium should be checked and maintained as a high normal level [13]. Temporary transvenous pacing is an effective way of controlling TdP. Pacing is particularly effective in pause-dependent long QT syndrome [14]. If all of these treatments are ineffective, defibrillator should be carried out. There are only 3 cases in the literature reporting an infective endocarditis presenting with long QT syndrome.

Sayar et al. [15] reported a case of prosthetic mitral valve Brucella endocarditis complicated with torsades de pointes in a 58-year-old woman. 

Yuan et al. [16] reported a long QT syndrome in extensive infective endocarditis complicating hypertrophic obstructive cardiomyopathy in a 52-year-old female.

 Irie et al. [17] described a QT interval prolongation andtorsadesdepointesinduced by propofol and hypoalbuminemia in a 70-year-old man presented with IE. 

This case is unusual in its presentation. The patient had a markedly prolonged QTc interval, and associated torsades de pointes and ventricular fibrillation as presenting scenario, and surprisingly the QT interval normalized after the thirtieth day of appropriate medical therapy, coinciding with the patient's clinical and hemodynamic stabilization.

## 4. Conclusion

This case exemplifies that TdP due to acquired long QT syndrome is a serious and potentially fatal complication in IE. Multiple factors including antimicrobial drugs are at an increased risk for the development of acquired long QTc syndrome. Physicians should therefore always maintain a high degree of clinical suspicion for the presence of long QT syndrome in patients with IE and should be aware of the QT-prolonging side effects of drugs they prescribe for these patients.
